# Foreign body mimicking malignancy in acquired dacryocystocele

**DOI:** 10.1002/ccr3.826

**Published:** 2017-02-03

**Authors:** Ahmad A. Mirza, Atheer F. Alsharif, Omar A. Elmays, Osama A. Marglani

**Affiliations:** ^1^ Department of SurgeryCollege of MedicineTaif UniversityTaifSaudi Arabia; ^2^ Faculty of Medicine and University HospitalKing Abdulaziz UniversityJeddahSaudi Arabia; ^3^ Department of Otolaryngology‐Head and Neck SurgeryAl‐Noor Specialist HospitalMakkahSaudi Arabia; ^4^ Department of Otolaryngology‐Head and Neck SurgeryKing Abdullah Medical ComplexJeddahSaudi Arabia; ^5^ Department of Otolaryngology‐Head and Neck Surgery and OphthalmologyFaculty of MedicineUmm Al‐Qura UniversityMakkahSaudi Arabia; ^6^ Head and Neck and Skull Base CenterKing Abdullah Medical CityMakkahSaudi Arabia

**Keywords:** Dacryocystocele, foreign body, granulation tissue, malignancy

## Abstract

A featured malignant‐like granulation tissue can be the only preoperative clinical clue of a concealed foreign body in the nasal cavity. Thus, endoscopic Dacryocystorhinostomy (DCR) should be completed with intraoperative nasal exploration to reveal non‐apparent foreign bodies that might be the underlying etiology of chronic dacryocystocele.

## Introduction

Dacryocystocele is a diffuse and centrifugal swelling of the lacrimal sac, which develops due to obstruction of lacrimal drainage system proximally and distally anywhere along the widespread canaliculi and nasolacrimal duct, respectively 1. It is typically congenital in origin and often presents in the first few months of life. An acquired dacryocystocele is extremely rare and commonly associated with a bluish swelling in the medial canthal areas and watery eyes (epiphora), which can be complicated by acute dacryocystitis, mucopurulent discharge, and periorbital cellulitis 2, 3. Causes of acquired dacryocystocele may include inflammation, neoplasm, facial trauma, or nasal surgery that mostly present unilaterally 1. To our knowledge, dacryocystocele has been unclearly acquired as a consequence of a previously existing foreign body occluding the nasolacrimal passages. So, to aid in the diagnosis and treatment of such a case, we will present a unique clinical vignette of dacryocystocele in a 57‐year‐old woman caused by a long‐standing nasal foreign body that promoted growing of granulation tissue, which behaves like a nasal malignancy.

## Case History/Examination

**Figure 1:**
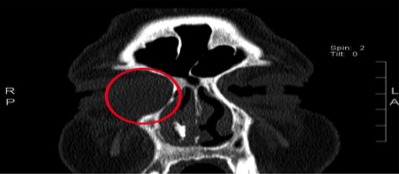
Preoperative coronal CT imaging of paranasal sinus showing dacryocystocele characterized by a low‐density, well‐defined cystic, fluid‐filled structure within the anteromedial aspect of the right orbit, adjacent to the nasolacrimal duct (circle).

A 57‐year‐old woman presented to our ENT clinic complaining of a spontaneous onset of right eye tearing, sensation of mucus at the back of her throat, and foul smell emanating from her nose for the past 8 years. She was otherwise healthy with no prior ocular history. On direct questioning, the patient recalled that she had pushed a rubber stopper from a perfume bottle up her nose when she was aged 8 years. Surprisingly, she does not remember whether this stopper was ever retrieved since then. A clinical examination revealed a palpable right medial canthal swelling that was diagnosed as dacryocystocele later on as illustrated in Figure 1.

## Differential Diagnosis, Investigations, and Treatment

**Figure 2:**
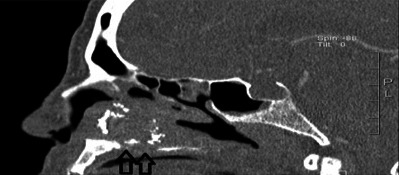
Preoperative sagittal CT imaging of paranasal sinus demonstrating multiple erosions of the hard palate (arrows).

**Figure 3:**
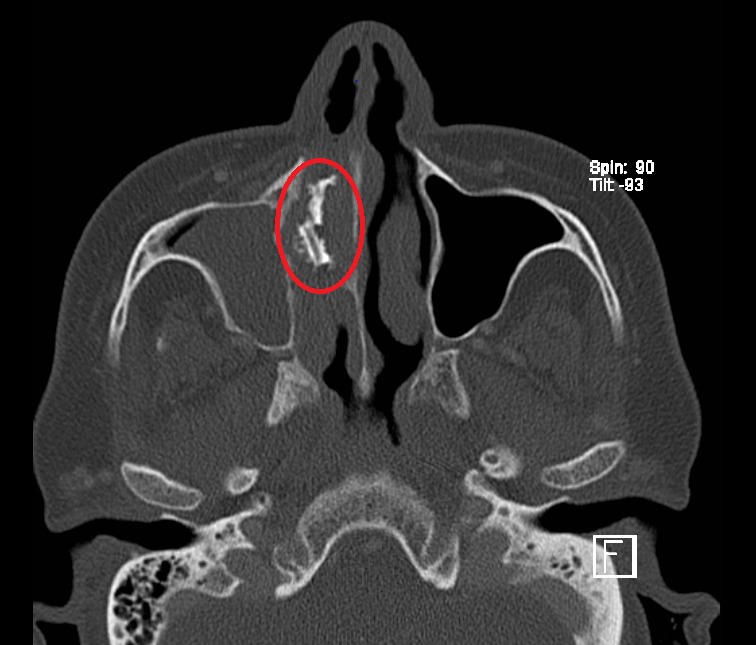
Preoperative axial CT imaging of paranasal sinus demonstrating a fragmented high‐density lesion within the underlying dacryocystocele. (circle).

Then, an endonasal endoscope was employed revealing a granulation tissue in the nasal cavity with a minimal amount of catarrh. However, no evidence of a foreign body was identified using this endoscopic mean. The patient was then investigated by utilizing CT paranasal sinuses, which has reported features of a mass in the right nasal cavity with multiple erosions in the floor of the hard plate and was associated with dense calcification. Wherefore, a suspicion of malignancy was significantly raised as shown in Figures 2, 3.

**Figure 4:**
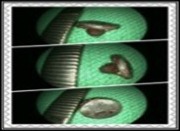
A 3 × 1 cm rubber foreign body that was found in the center of the granulation tissue.

The patient underwent right nasal cavity endoscopic exploration under general anesthesia, and the mass, which was surrounded by granulation tissue, was excised completely. Interestingly, upon removal of the mass, a rubbery object was identified within the granulation tissue and measured at 3 × 1 cm (Fig. 4). Subsequently, the patient underwent endoscopic dacryocystorhinostomy (DCR) to restore the flow of tears from the lacrimal sac into the nose. A gush of yellowish, mucoid fluid was drained from the distended lacrimal sac. Histological examination revealed an inflammatory reaction without any potential neoplastic characters.

## Outcome and Follow‐up

Afterward, the patient had an uneventful recovery, and the mass has not recurred within 3 years of follow‐up and no complications have been encountered.

## Discussion

In most cases, dacryocystoceles transpire congenitally while only few cases have been reported for the acquired form 1, 2, 4, 5, 6. Many underlying etiologies have been associated with acquired dacryocystoceles including severe inflammation, surgery, radiation therapy, and also malignant tumors originating from medial canthal or sinonasal regions disrupting the nasolacrimal duct and causing dacryocystocele 3, 4, 7. Pathogenic mechanism knowledge of dacryocystocele due to a foreign body is unclear. Nevertheless, the pathogenic mechanism related to congenital dacryocystocele can present the basis for understanding the occurrence in adults. The occurrence of congenital dacryocystocele is usually caused by failure of canalization of lacrimal pathway or a membranous block at the valve of Hasner. The obstruction is functional in many patients with dacryocystocele, in which no signs of anatomical obstruction exist along the lacrimal apparatus as evidenced by history and clinical examination. In cases of dacryocystocele in adults, the same mechanism can be involved 8. In our case, the patient was symptomatic, medial canthus swelling was detected, and finally, uncomplicated dacryocystocele was diagnosed clinically and the granulation tissue acquisition might be the underlying mechanism of developing this acquired form of dacryocystocele.

Moreover, despite using a nasal endoscope, an important adjunct in the evaluation of dacryocystocele 9, no clear evidence of foreign body was identified. Instead, a mass of granulation tissue was found with a minimal amount of catarrh.

Scholars have investigated imaging features of dacryocystoceles and demonstrated that CT exposed various features including cystic medial canthal mass, dilatation of the nasolacrimal sac, fluid‐filled structure with slight rim enhancement, no solid components and so forth 4, 10. As a result, we carried out a CT of paranasal sinuses that demonstrated a well‐defined cystic, fluid‐filled structure within the anteromedial aspect of the right orbit, adjacent to the nasolacrimal duct. Moreover, we have found a mass in the right nasal cavity that associated with worrisome characteristics including erosions of the hard plate and dense calcification, which suggested the possibility of an underlying malignancy. A study published in 2007 revealed that a high index of suspicion for sinonasal malignancies, which can result in dacryocystocele, should be paid during the radiological evaluation by identifying, initially, the CT findings of bone destruction and erosions 4. No mention was found in the literature about specific radiological features for foreign body‐induced granulation tissue or whether it could take on a malignancy‐like appearance causing dacryocystocele. One case of immunoglobulin G4‐related sclerosing disease, an inflammatory syndrome, was, however, found in the English medical literature and that mimics malignancy on initial CT findings 11. On the other hand, malignant tumors causing dacryocystocele have quite distinct features; more importantly contrast enhancement on CT acquisition, which reduces their likelihood to be mistaken with benign causes 4, 12. In the previously mentioned case of immunoglobulin G4‐related sclerosing disease, images were enhanced in a similar way to malignant lesions 11. MRI is used as a complementary investigation for paranasal masses 13, as aggressive bony malignant findings are generally well identified on CT 14. In addition, CT occupies an important place in predicting prognosis and assessing mass extent 14.

In this case, we conducted a right nasal cavity endoscopic exploration to remove the granulation tissue containing the foreign body and rule out the underlying malignancy. Tabatabaie et al. reported a forging body, which was surrounded by a granulation tissue. However, the object was located in the lacrimal system and did not show any susceptibility to malignancy 15.

After establishing the diagnosis using lacrimal excretion test as well as radiological assessment, the result of suitable surgical procedure must be useful. Acquired dacryocystocele can be managed by external or endoscopic approaches with equal success rates 16. However, endoscopic DCR is superior due to the avoidance of facial scarring, preservation of the pumping mechanism of the orbicularis oculi muscle, minimal postoperative hematoma, and faster recovery 16. In some cases, DCR can be integrated with a placement of silicone tube or dacryocystectomy. However, they are not necessary 2, 6. In our case, we utilized the endoscopic approach of DCR because it was a practical approach when it comes to restoring the flow of tears from the lacrimal sac into the nose 5.

## Conclusion

By and large, a long‐standing foreign body inducing acquired dacryocystocele should be suspected clinically in a patient with a remarkable history along with presence of granulation tissue. The use of a flexible endoscopic technique clinically along the nasal passage is not highly accurate for detecting an underlying foreign body provoking dacryocystocele. In such cases, CT imaging of paranasal sinuses could be misinterpreted in diagnostic purposes as some features of underlying malignancy might be identified in those patients who develop overlying granulation tissue. Appropriate surgical approach relies on accurate preoperative evaluation of the paranasal mass, which can be ensured by CT imaging in majority of cases.

## Ethical Consideration

A patient's consent was sought to report this novel presentation.

## Authorship

AAM: literature review, novelty determination and case report writing. AFA: literature review and writing the report. OAE: Case evaluation. OAM: Case evaluation, novelty specification and report review with multiple corrections.

## Conflict of Interest

The authors declared no potential conflicts of interest with respect to the research, authorship, and/or publication of this article.
